# Planning Fail-Safe Trajectories for Space Robotic Arms

**DOI:** 10.3389/frobt.2021.710021

**Published:** 2021-11-30

**Authors:** Oliver Porges, Daniel Leidner, Máximo A. Roa

**Affiliations:** ^1^ Agile Robots, Munich, Germany; ^2^ Institute of Robotics and Mechatronics, German Aerospace Center (DLR), Wessling, Germany

**Keywords:** manipulation planning, robotic arms, fail-safe trajectories, fault-tolerant manipulators, space manipulator

## Abstract

A frequent concern for robot manipulators deployed in dangerous and hazardous environments for humans is the reliability of task executions in the event of a joint failure. A redundant robotic manipulator can be used to mitigate the risk and guarantee a post-failure task completion, which is critical for instance for space applications. This paper describes methods to analyze potential risks due to a joint failure, and introduces tools for fault-tolerant task design and path planning for robotic manipulators. The presented methods are based on off-line precomputed workspace models. The methods are general enough to cope with robots with any type of joint (revolute or prismatic) and any number of degrees of freedom, and might include arbitrarily shaped obstacles in the process, without resorting to simplified models. Application examples illustrate the potential of the approach.

## 1 Introduction

Robotic manipulators are a convenient tool for deployment in dangerous and hazardous environments for humans, for applications such as planetary exploration, on-orbit servicing, de-orbiting of uncooperative targets, and hazardous material handling in nuclear or chemical disaster/waste sites. High cost and low to none maintenance possibilities place strong demands on reliability for robots deployed in such scenarios. The economic cost of a failure in those environments is usually very high, as analyzed in Ellery et al. (2008). Thus, one of the major concerns in these situations are the post-failure capabilities of the robotic manipulator. Even though the redundancy of electronics and mechanics of the joints significantly reduces the risk, the use of a redundant manipulator adds another layer of safety for the operation. This paper is focused on the description of methods and tools for risk analysis and fault-tolerant path planning to ensure the post-failure task execution for such robotic manipulators.

Most of the literature on safety-critical operations of robotic manipulators considers that if a failure occurs, it would be a locked joint. A free-swinging joint is another possible failure, but unlikely to occur given the considerations employed in the mechatronic design of joints. Early works on fault-tolerant path planning or task design establish a link between robustness to failures and kinematic dexterity based on the minimum singular value of the post-failure Jacobian, as presented in Lewis and Maciejewski (1994). This post-failure dexterity is referred to as the *kinematic failure-tolerance measure* (*kfm*). The *kfm* values are analyzed throughout the configuration space (C-space) in order to identify configurations of optimal fault-tolerance. An inverse of the optimal *kfm* is used to track an end-effector path to anticipate failures and guarantee post-failure task execution. The methods are demonstrated on a planar manipulator with three DoF (Degrees of Freedom) realizing a 2-DoF task without obstacles.

Global methods for fail-safe path planning have also been considered. A global path planning approach for post-failure operation by using a redundancy resolution algorithm was proposed in Paredis and Khosla (1996). At each point along a desired end-effector path, a set of acceptable fault-tolerant configurations is estimated. A connectivity graph is constructed from this set, capturing the pre-image topology along the path. In case of failure, a graph search discovers alternative trajectories to reach the goal end-effector pose. The approach is demonstrated on a 4-DoF robot. Another global approach in Lewis and Maciejewski (1997) discusses the conditions of existence of fault-tolerant regions of operation in C-space around the start and goal poses. The authors overcome computational limitations by computing off-line the self-motion manifolds, which are used to modify the task by changing the goal pose so that larger self-motion manifolds are used during the task execution. The path generation method artificially imposes restrictions on joint limits to avoid possible failure scenarios. The analysis and proof of existence of a fault-tolerant path is done using intersections of self-motion bounding boxes in C-space, representing safe ranges for operation. The self-motion manifolds are discovered through a sampling approach, and they are explored by executing a spiral motion along the manifold to determine its boundary and connectivity. Joint configuration constraints on fault-tolerant motion are derived from the intersection of start and goal self-motion bounding boxes. Potential collisions of the robot are not included in this method.

A hierarchical method for failure analysis using a least-constraint framework is presented in Ralph and Pai (1997). Here, the main task is to reach the goal, and the secondary task is resolving the null-space configuration to maximize the utility of redundancy. The task is formulated as a set of constraints rather than using waypoints, and introduces a performance measure called *longevity*, which reflects the connectivity of the C-space after a given failure. A dynamic programming task computes the *longevity* and searches for a post-failure recovery path using pre-computed recovery strategies (*ease of recovery*) required to keep the functionality of the robot. The method is demonstrated on a 4-DoF robot without obstacles; an extension of this framework to a 5-DoF robot is presented in Ralph and Pai (1999). A fault-tolerant path planning method for a 7-DoF manipulator is presented in Jamisola et al. (2006). A locked-joint failure at a start configuration defines *failure hyperplanes*. Intersections of these hyperplanes with the goal pose self-motion manifold define a failure hypercuboid. A web of paths is generated from the start configuration to a point on the goal pose self-motion manifold using monotonic paths. After checking continuity and collisions, an obstacle-free web of paths is created, defining a *failure surface*. While the manipulator configuration stays on the failure surface, it is guaranteed that the goal end-effector pose can be reached after any failure occurs. A measure for global failure tolerance is derived based on the joint range excursion in the self-motion manifold. The maximization of the size of the self-motion manifolds was recently proposed in Alkmarkhi and Maciejewski (2019), by using singularities to identify where large manifolds might exist.

In summary, previous works have identified the necessary conditions under which a redundant manipulator can fulfill a task after a joint failure, exploiting links between dexterity, manipulability and robustness to failures. The methods are commonly based on the analysis of pre-image and self-motion manifold estimation using discrete sampling and off-line computations of certain aspects of the algorithms (to overcome the high computational costs). The constraints derived from such analysis are used for path verification and planning. Several measures of robustness to failure were introduced. Obstacle avoidance, if addressed, relies on projection of primitives in the C-space. The path-planning approaches are typically augmented with discrete sampling or with a null-space controller. The analysis of failure robustness is performed mainly on a case by case basis. Some works have considered as well a global overview, as the one provided here, but generic considerations of obstacles in the environment and coupling of the methods with path planning algorithms are scarce in previous works.

This paper builds upon the necessary conditions for existence of a fault-tolerant path and presents a new approach to fail-safe path planning and task design. Our framework is based on a global workspace overview, which allows a direct link between the end-effector pose and its pre-image bounds in C-space. Not relying on local exploration, all self-motion manifolds (which compose the pre-image) for fault-tolerant path planning are revealed, regardless of the current joint configuration. Pre-image bounds are pre-computed in the offline phase and used to generate a model that provides information about robot redundancy within the workspace and the effects of failures on the end-effector positioning ability. This information is later used to provide the required constraints for the on-line fault-tolerant path planning process. Thus, the method is applicable to arbitrary kinematic designs and to large DoF systems without further adaptation. The environment around the manipulator (e.g. the satellite body) can also be considered in the workspace model and in the computation of the self-motion manifolds. For this purpose, collision detection does not rely on C-space projection but it uses directly the geometry of the environment, therefore, it is possible to use any common collision detection method within the approach. Our implementation uses a point-to-primitive check, which makes the method suitable for online applications utilizing depth sensor input directly. The framework is applied to different scenarios that demonstrate the potential of the proposed approach.

## 2 Reachability and Capability Maps

A redundant manipulator has at least one DoF more than the number of DoF required for the task at hand. In this paper, a set of joint values for the manipulator is denoted as $\theta \in R^{N_{dof}}$ (with N_dof_ representing the number of joints), which corresponds to a point in the C-space for the manipulator. Joint values *θ* are bounded by a set of joint limit pairs denoted as $Q=\left\{\theta_{min},\theta_{max}\right\}$ . A forward kinematics function maps the C-space configuration *θ* to a task space pose ξ ∈ *SE* (3). An inverse kinematics function maps *ξ* → θ, and it typically provides multiple solutions for redundant manipulators. As there can be many *θ* mapping to one particular *ξ*, self-motion manifolds are present, as discussed in Burdick (1989).

The workspace W of a manipulator refers to every possible *ξ* that can be reached with a configuration *θ* within the joint limits *Q*. The workspace W can be represented in a discrete way through the reachability map. It captures all poses reachable by the robot’s end-effector frame with respect to the robot base. These maps were initially introduced in Zacharias et al. (2007) and Diankov (2010), and have been used in multiple applications including workspace analysis and robot design in Porges et al. (2015), pre-filtering of inverse kinematics queries in Porges et al. (2014), robot base positioning as in Zacharias et al. (2009b); Vahrenkamp et al. (2013), bi-manual manipulation planning in Sundaram et al. (2016); Vahrenkamp et al. (2009), path generation or validation in Zacharias et al. (2009a) and humanoid robot foot-step planning in Werner et al. (2016).

**FIGURE 1:**
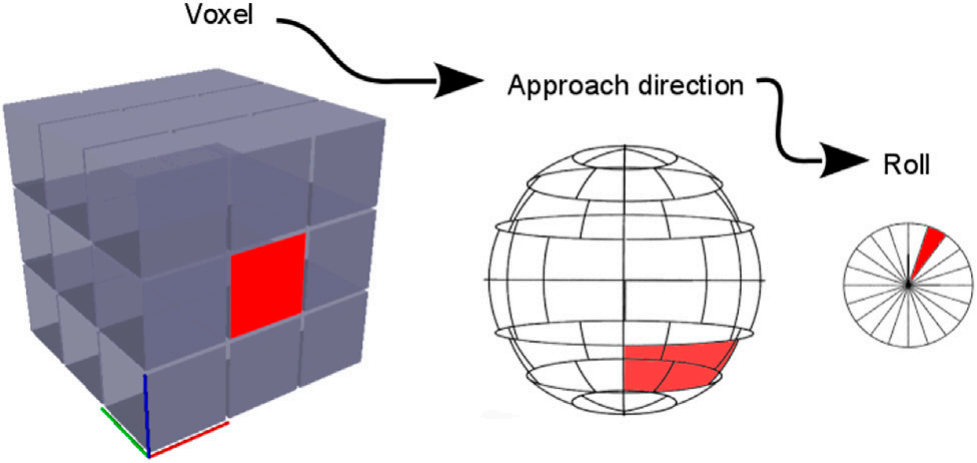
Hierarchical discretization of the end-effector pose *ξ* for efficient generation of the reachability map. The Cartesian space is discretized with voxels. Each voxel has an associated virtual sphere that discretizes the possible approach directions (pitch and yaw). Each surface bin in this virtual sphere has in turn an associated discretization for the roll direction.

To obtain the reachability map, the workspace is discretized in a hierarchy that decomposes the pose ξ ∈ SE (3) into a translation and a rotation, ξ → (t, R), where t ∈ R^3^, R ∈ SO(3). Considering the Cartesian position *t* → *x*, *y*, *z* and Euler angles *R → α, β, γ* (roll, pitch, yaw), the mapping functions f(t) → V_i_ and g(R) → v_i,p_ discretize the pose *ξ* using *i*, p ∈ N, where V_i_ is the i-th voxel and *v*_*i*,*p*_ is a particular bin value of voxel *V*_*i*_. An illustration of the discretization mechanism is presented in Figure 1. The workspace is discretized with voxels of suitable size. Each voxel has an associated virtual sphere that discretizes the end-effector pointing direction (pitch and yaw, *β* and *γ* Euler angles) within each voxel. Each discretized pair of *β*, *γ* has an associated grid for discretizing the roll angle *α*. The reachability map stores binary values for each bin, where a bin represents a small range of end-effector poses in *SE* (3): The stored value is one for reachable or zero for unreachable bins. The level of detail of a reachability map is then represented by three quantities, voxel size (e.g. 0.025m), number of approach directions (e.g. 200) and number of roll bins (e.g. 30, thus mapping 200 × 30 = 6,000 orientations in each voxel).

This paper exploits two fundamental functionalities of reachability maps: pose existence queries and interpreter functions. A query to a map is denoted as W(ξ) , and performs the mapping of pose *ξ* into a bin and retrieves its binary value, which indicates whether the pose is reachable or not. The speed of this query process is what makes the reachability maps suitable for on-line applications on robots. The current implementation from Porges et al. (2014) handles typically about 2000 queries per millisecond. Interpreter functions help to visualize the workspace model data based on a desired property. A commonly used interpreter function called reachability index *R* summarizes the results for all discretized orientations inside a voxel in one metric, thus enabling the workspace visualization. This index is defined by

$$R\left(V_{i}\right)=\frac{\sum_{p=0}^{n_{b}}v_{i,p}}{n_{b}}$$ (1)

Where *n*_*b*_ is the number of all discretized orientations inside each voxel (approach directions times roll directions). The range of the reachability index is $R\left(V_{i}\right)\in \left[0,1\right]$ , from totally unreachable (*R* = 0) to 100% reachable orientations (*R* = 1) in a voxel.

**FIGURE 2:**
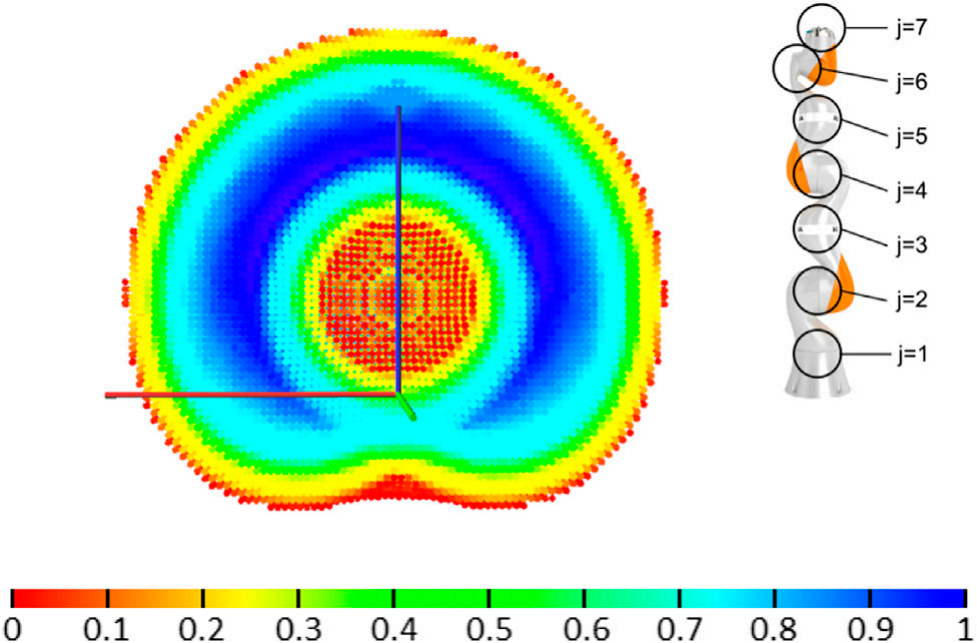
Cross-section of the capability map for a KUKA iiwa robot, with seven DoF (joints *j* are marked in the figure). The HSV color scale encodes the reachability index *R* given by Eq. 1. Map and robot are plotted at the same scale.

The reachability index reflects a local dexterity in the task-space, as it indicates the ability of the manipulator to reach different position and orientations of the end effector within the local neighborhood of the current pose. Note that reachability strictly refers here to the ability to reach all possible orientations of the end effector at a given point in the manipulator workspace, as a difference to the manipulability measure derived from the Jacobian condition number in Yoshikawa (1985), which describes the ability of the manipulator to move and apply forces in arbitrary directions, as described in Patel and Sobh (2015). Further comparison on reachability and manipulability is provided in Zacharias (2012). A 3D visualization of voxels with a color-coded reachability value is called a capability map, as illustrated in Figure 2 using a HSV color scale: Regions with low reachability are colored in red, while high reachability regions are colored in blue. To provide a sense of scale, the capability maps shown in this paper always include a reference frame, with axis of 1*m* length, depicting the robot base position and orientation. Generation methods, performance and prediction accuracy of these maps are discussed in detail in Porges et al. (2014).

## 3 Failure Maps

Since the reachability maps depend on the kinematic design of the manipulator, they are usually computed off-line, and the information can later be queried for online processes. In case that an arbitrary joint of the robot is locked, a set F containing a large number of reachability maps can be generated to represent the effect of the locked joint on the robot workspace. Let $W_{j,l}$ be a reachability map where *l* is the locked position of the *j*-th joint,

$$F=\left\{W_{j,l}|j\in \left(1,N_{dof}\right),l\in \left(l_{min},l_{max}\right)\right\}$$ (2)

Where l_min_, l_max_ ∈ Q_j_ are the joint limits of the locked joint. For illustrating the methods in this paper, the 7-DoF KUKA iiwa robot^1^ is used as a prototypical example. To generate F , each joint is locked, one at a time, at different successive positions within the joint limits using a given resolution. This paper uses a resolution of one degree, which leads to generating 2097 reachability maps. The maps are generated using forward kinematics, which provides the least amount of false positive values in prediction of pose reachability, as discussed in Porges et al. (2014).

**FIGURE 3:**
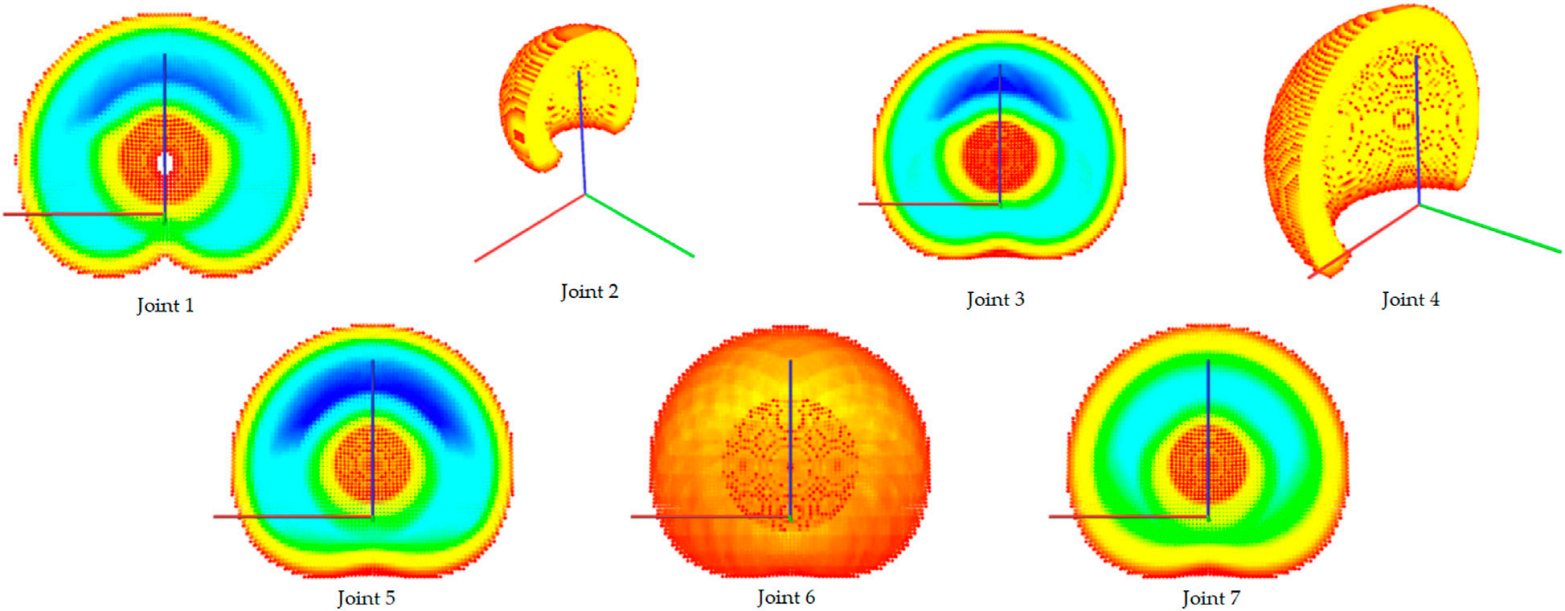
Capability maps for the KUKA iiwa robot when each joint is locked at its zero position while all the other joints are fully operational. The cross-section is displayed using the same scale and the same cutting plane (XZ), but the point of view is changed in some maps to provide a better 3D visualization. Note that a failure in joint 2 or 4 significantly affects the workspace volume, while a failure in joints 6 or 7 reduces dexterity throughout the workspace (compare to the original capability map without failures in Figure 2).

Joint locks reduce the capabilities of the manipulator. They shrink the post-failure workspace and reduce the manipulator’s dexterity. Figure 3 illustrates the influence of a potential failure at different joints locked at zero position on the workspace of the KUKA iiwa robot.

Some workspace locations are more affected than others by joint failures. To gain a full overview for assessing the risk, workspaces from the set F are merged into one failure map $W_{f}$ by

$$W_{f}=\overset{N_{dof}}{\underset{j=1}{\sum }}\overset{l_{max}}{\underset{l_{min}}{\sum }}W_{j,l}$$ (3)

**FIGURE 4:**
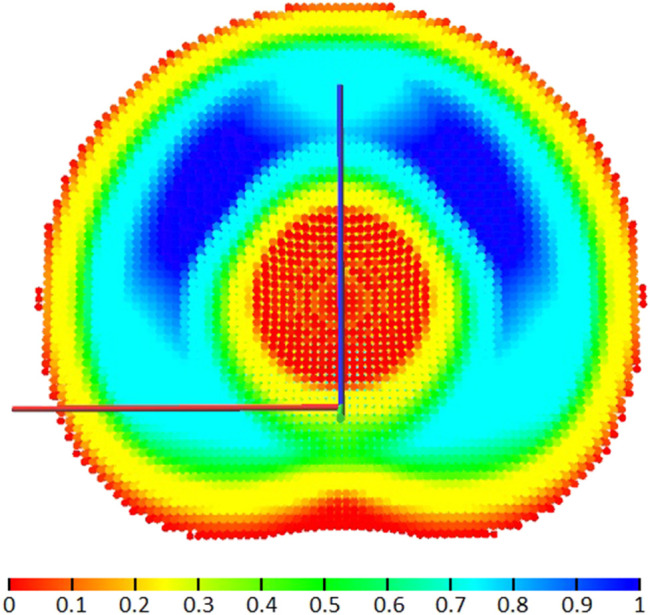
Cross-section of the failure map $W_{f}$ for the KUKA iiwa robot.

Two reachability maps can be merged as described in Sundaram et al. (2016): each bin v_i,p_ ∈ N of the resulting map $W_{f}$ holds an integer value reflecting the number of maps from the set F that identify this particular bin as reachable. A new interpreter function called failure index *F*(*V*_*i*_) is computed in an analogue manner to the reachability index, and defined as

$$F\left(V_{i}\right)=\frac{\sum_{p=1}^{n_{b}}v_{i,p}^{f}}{n_{m}*n_{b}}\;v_{i,p}^{f}=\overset{n_{m}}{\underset{k=1}{\sum }}v_{i,p}^{\left(k\right)}$$ (4)

where $v_{i,p}^{\left(k\right)}$ is the *p*-th bin value of voxel *i* in reachability map *k*, and *n*_*m*_ represents the number of maps generated, i.e. the number of points used to discretize the joint ranges *Q*. Figure 4 shows the cross-section of the failure map for a KUKA iiwa. With the HSV color scale, red areas are less robust to a general joint failure, while blue areas are reachable in most failure cases. The combined failure map $W_{f}$ is used for identifying Cartesian positions of lower and higher risk due to joint failures, so that locations with higher risk are avoided when performing critical operations.

The map in Figure 4 appears intuitive due to its symmetry, as it is generated for a robot moving in a collision-free environment. For a real use case, the workspace models can be generated considering collisions with the known environment, e.g. the mobile base of the manipulator or the satellite for a space robot arm, as presented in Porges et al. (2015). A small obstacle can cause large reachability reductions away from the obstacle’s vicinity.

The failure index defined in (4) summarizes in a single index the influence of the joint failure across all orientations. The index can be tuned to study failures along a specific subset of orientations, or just in a single orientation, by changing the limits of the sum in (4).

Individual bin values in $W_{f}$ indicate the robustness of the end-effector pose. The bin value *v*_*i*,*p*_ is calculated as a sum of all maps from F containing the pose *ξ* associated with it, therefore, v_*i*,*p*_ quantifies the pre-image footprint within *Q*. In general, the larger the bin value v_*i*,*p*_, the more failure-tolerant behavior can be achieved. For example, particular values *v*_1_ = 101 and *v*_2_ = 1622 mean that the pose associated with *v*_1_ is tolerant to failure in 4.8*%* of the cases, while *v*_2_ is failure-tolerant in 77.3*%* of the 2097 discretized cases.

**FIGURE 5:**
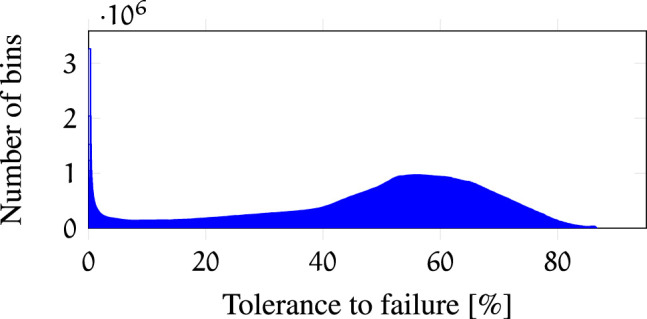
Histogram of bin values of all mapped poses *ξ* in $W_{f}$ .

**FIGURE 6:**
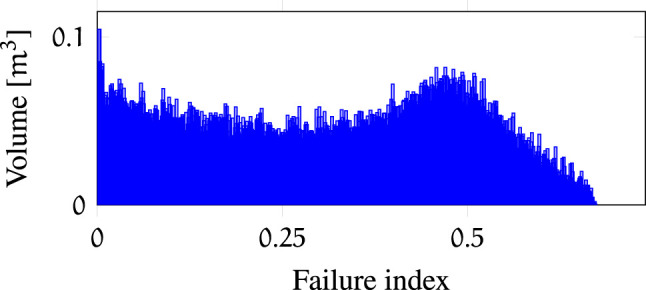
Histogram of failure index *F*(*V*_*i*_) values in $W_{f}$ .

Other measures derived from $W_{f}$ help to evaluate the robot kinematics. A histogram of the bin values is presented in Figure 5. The maximum bin value in $W_{f}$ is 1813 out of 2097 original joint failure maps, therefore, the maximum possible robustness of a particular pose in the workspace is 86.46*%* and the highest failure index of a voxel is 0.673. The area under the curve is correlated to the 6-dimensional volume that indicates the portion of task space robust to failures. A histogram of the failure index is presented in Figure 6. The area under the curve represents the 3-dimensional volume (correlated to the number of voxels) that indicates the portion of the workspace robust to failures. These histograms are useful for instance to compare different kinematic designs and verify which ones provide larger volumes of high tolerance to failures.

**FIGURE 7:**
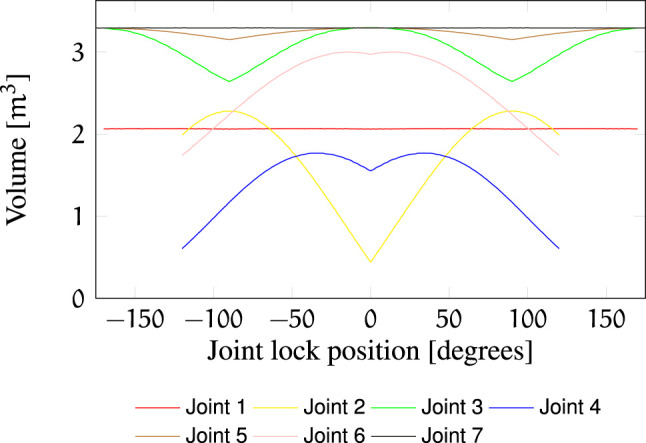
Post-failure workspace volume of $W_{j,l}$ as a function of *l*. The nominal failure-free volume is 3.292 m^3^.

**FIGURE 8:**
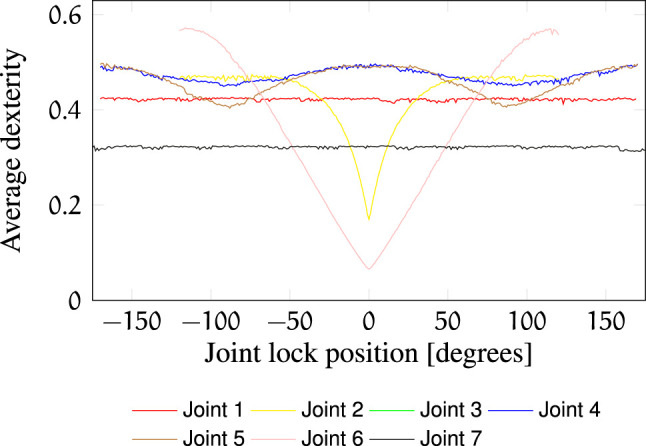
Post-failure average dexterity in $W_{j,l}$ as a function of *l*. The average dexterity for the nominal failure-free workspace is 0.578.

Additional indicators of failure robustness are the post-failure workspace volume (Figure 7) and the post-failure average dexterity (Figure 8). The post-failure workspace volume shown in Figure 7 helps to identify joint value ranges in which a joint failure is dangerous. In this case, joint 2 around a joint value of 0 is critical. A cross section of this particular post-failure capability map is shown in Figure 3. The post-failure workspace volume has almost no change for failures in joints 1 and 7. On the other hand, the average dexterity, shown in Figure 8, is significantly reduced for joints 2 and 6 around the joint value 0. A cross section of the capability map for these failures is shown in Figure 3, for the joints locked at zero position. When designing the kinematic structure, these indicators can be used to avoid concentration of risks in one zone of the C-space. How critical are these C-space locations can only be evaluated for a particular task specification.

## 4 Failure Diagrams

Previous sections described a metric for failure robustness, and a global overview of the redundancy of the manipulator kinematics with insight into risks related to failures of single joints. In order to guide the task design or to plan fault-tolerant paths, more insight into the C-space is required. For this purpose, a new tool called failure diagram, denoted as D(*ξ*), is introduced. It is a function of the end-effector pose *ξ*, and can be generated from the set F . The set F is queried with additional parameters that specify the joint and its locked position $W_{j,l}\left(\xi \right)$ . The failure diagram *D*(*ξ*) is defined as

$$D\left(\xi \right)=\left\{W_{j,l}\left(\xi \right)|j\in \left(1,N_{dof}\right),l\in \left(l_{min},l_{max}\right)\right\}$$ (5)

**FIGURE 9:**
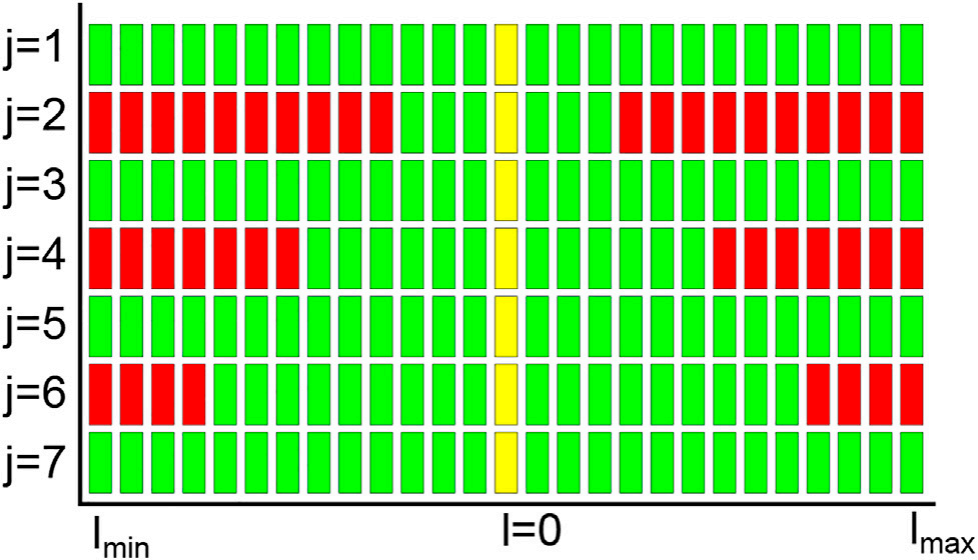
Normalized failure diagram for a given pose ξ; the ranges of the seven joints *j* have been normalized for illustration purposes (naturally, each joint has its own joint limits, which do not necessarily coincide). Yellow cells represent the current joint configuration, in this case, the upright position (all joints at zero). Green/red cells represent reachability maps $W_{j,l}\left(\xi \right)$ that do/do not contain the pose ξ.

**FIGURE 10:**
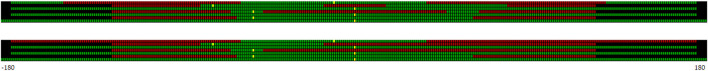
Failure diagrams with one highlighted joint configuration (in yellow). Each row represents one joint (similar to Figure 9). The diagrams are not normalized as in Figure 9, i.e the joint range spans from -180 to +180 deg. Regions that are out of the joint limits are represented as black cells. **Top:** Failure diagram showing all C-space manifold footprints. **Bottom:** Failure diagram showing only the C-space manifold footprint relevant for the current configuration.

An illustrative failure diagram is presented in Figure 9. Each horizontal row represents one joint within its range of motion. Every colored cell corresponds to a reachability map $W_{j,l}$ with *j*, *l* according to the plot axes. The color of the cell indicates the reachability of the pose ξ in the referenced reachability map, green for reachable and red for unreachable. The pattern of the diagram shows continuous ranges of green and red cells in each joint, hereafter called allowed and forbidden ranges, respectively. This pattern is the pre-image footprint of ξ in Q. Multiple allowed ranges within one row (joint) indicate disconnected manifolds of the pre-image. To illustrate this point, a typical failure diagram (non-normalized) for the KUKA iiwa is presented in Figure 10 top.

Number and structure of distinct self-motion manifolds within a pre-image of a redundant manipulator are examined in Burdick (1989). Two self-motion manifolds are either (C-space) disconnected or connected through a singularity. Each self-motion manifold is continuous in its own. As a result, if a joint is moved within its allowed ranges on the diagram D(ξ), it is guaranteed that the pose ξ will be post-failure reachable. If any joint value falls outside the allowed ranges and a failure occurs there, the pose of D(ξ) is guaranteed to be unreachable. Transitioning of one joint value between two allowed ranges is, therefore, not possible. The combination of allowed ranges (one for each joint) is not arbitrary. A valid combination of allowed ranges is determined by the existence of joint values θ for pose ξ that lie within these ranges, i.e. the allowed range selection is derived from the pre-image of ξ. This selection process is illustrated in Figure 10. The joint values in Figure 10 (top, indicated in yellow) are from the pre-image of ξ for which this diagram was generated. Based on this robot configuration, a selection of allowed ranges for fail-safe operation is shown in Figure 10 (bottom). Note that this failure diagram is obtained starting from capabilty maps, which have some chosen discretization level. In our case, we are using a voxel size of 0.025 m, with 200 approach directions and 30 roll bins. The failure diagram is then the pre-image of all the poses that fall into the bin containing the desired pose ξ. This tolerance to reaching the desired pose is of course task-dependent, and should be set by the user. To increase numerical accuracy, a finer resolution for discretization should be used, which leads to higher computational costs.

The reachability map arising from D(ξ) is denoted by W(D(ξ)) . The generation pipeline is described by

θ→ξ→D(ξ)→W(D(ξ)) (6)

**FIGURE 11:**
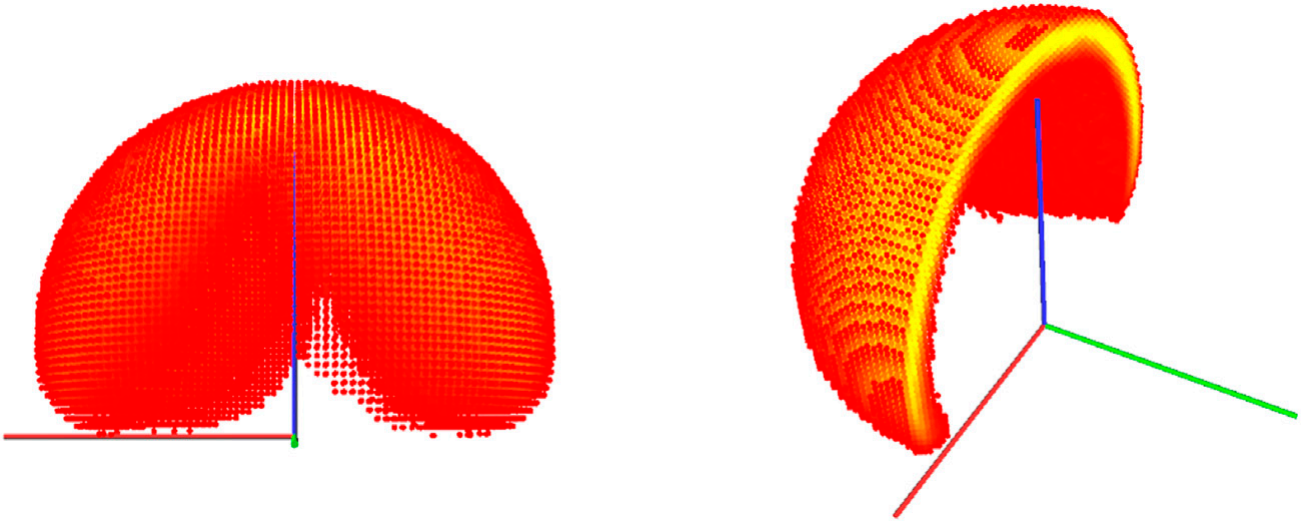
Capability map spanned from the failure diagram in Figure 10 **top**, and cross-section of the same map. It corresponds to the post-failure return-safe workspace for all pre-image manifolds.

**FIGURE 12:**
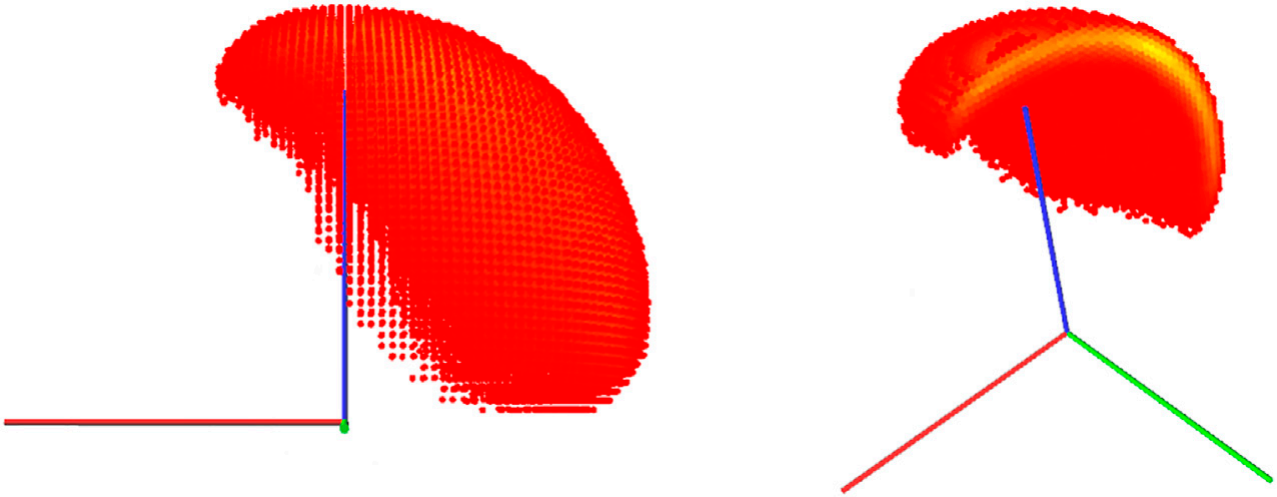
Capability map spanned from the failure diagram in Figure 10 **bottom**, and cross-section of the same map. It corresponds to the post-failure return-safe workspace only for the relevant self-motion manifold.

Given a desired configuration θ, the pose ξ is computed. The failure diagram D(ξ) for this pose is later used to generate the reachability map W(D(ξ)) . To illustrate the pipeline, Figure 11 shows W(D(ξ)) generated from the failure diagram of Figure 10 top. It contains all poses of the end-effector considered fail-safe with respect to the pose associated with the diagram D(ξ). These poses can be reached only if the robot configuration stays within a set of allowed joint ranges (one for each joint). Figure 12 shows the associated reachability map W(D(ξ)) for those poses, which are a subset of Figure 11. For practical reasons, the design process described in Eq. 6 starts from configuration θ.

During task design, we start with an arbitrary inverse kinematics solution for reaching the desired pose ξ. Failure diagram D(ξ) reveals all allowed ranges, and the robot configuration identifies the valid ranges of operation for each joint. W(D(ξ)) spanned from these identified ranges describes the task space for fail-safe operations. If a different combination of allowed ranges is desired (e.g. to move to a range with larger footprint in Q), the existence of such θ that selects the desired ranges has to be verified. Note that a post-failure path existence is guaranteed from an arbitrary pose $\xi_{a}\in W\left(D\left(\xi \right)\right),\theta \in D\left(\xi \right)$ to ξ and not vice-versa. The spanned workspace W(D(ξ)) is therefore called post-failure return-safe (*pfrs*) workspace.

Post-failure reachability can be guaranteed for multiple poses simultaneously. Let us have two end-effector poses *ξ*_1_ and *ξ*_2_ and their respective failure diagrams *D* (*ξ*_1_) and *D* (*ξ*_2_). An operator *D* (*ξ*_1_) & *D* (*ξ*_2_) is defined analogically to the logical operator &, where green cells represent the logical value one and red cells represent the value zero. The result of the operation & is a merged failure diagram *D* (*ξ*_1_& *ξ*_2_). A failure-tolerant path from *ξ*_1_ to *ξ*_2_ exists if the diagram *D* (*ξ*_1_ & *ξ*_2_) contains an allowed range for each joint. Similar to the previous case, a new reachability map is spanned as $W\left(D\left(\xi_{1}\&\xi_{2}\right)\right)$ . Such a map represents a safe workspace to both poses *ξ*_1_ and *ξ*_2_, which is significantly smaller with respect to the individual *pfrs* workspaces $W\left(D\left(\xi_{1}\right)\right)$ and $W\left(D\left(\xi_{2}\right)\right)$ . If the robot configuration stays within the boundaries of *D* (*ξ*_1_& *ξ*_2_), *ξ*_1_ and *ξ*_2_ are guaranteed to be post-failure reachable, i.e., a path between *ξ*_1_ and *ξ*_2_ will exist after any single joint failure.

## 5 Applications

To demonstrate the design and planning capabilities of the presented tools, three different use cases are discussed: online grasp selection, path planning, and base positioning for a robot.

### 5.1 Online Grasp Selection

The increasing amount of orbital debris is a major concern for space missions nowadays. Many dysfunctional satellites are occupying valuable orbits, and potential collisions with them might create more dangerous debris. Robotic manipulators could be the key technology to enable de-orbiting of uncooperative targets like ENVISAT, as discussed in Jaekel et al. (2015). On-orbit servicing is a promising technology to try to keep the orbits clean. Manned on-orbit service missions helped to keep the Hubble telescope afloat and functional, however, at a very high cost. Robotic manipulators could provide the means of servicing and refueling satellites at a reasonable cost, thus prolonging their lifetime in orbit. In a de-orbiting or servicing mission for satellites, a servicer satellite chases a target, or client satellite. The goal is to capture the target with the manipulator and perform a docking maneuver with the servicer. The target is not necessary cooperative, as it can be tumbling at high rotational velocities. High tumbling velocity and/or significant mass of the target lead to a high risk of damaging the manipulator on contact.

**FIGURE 13:**
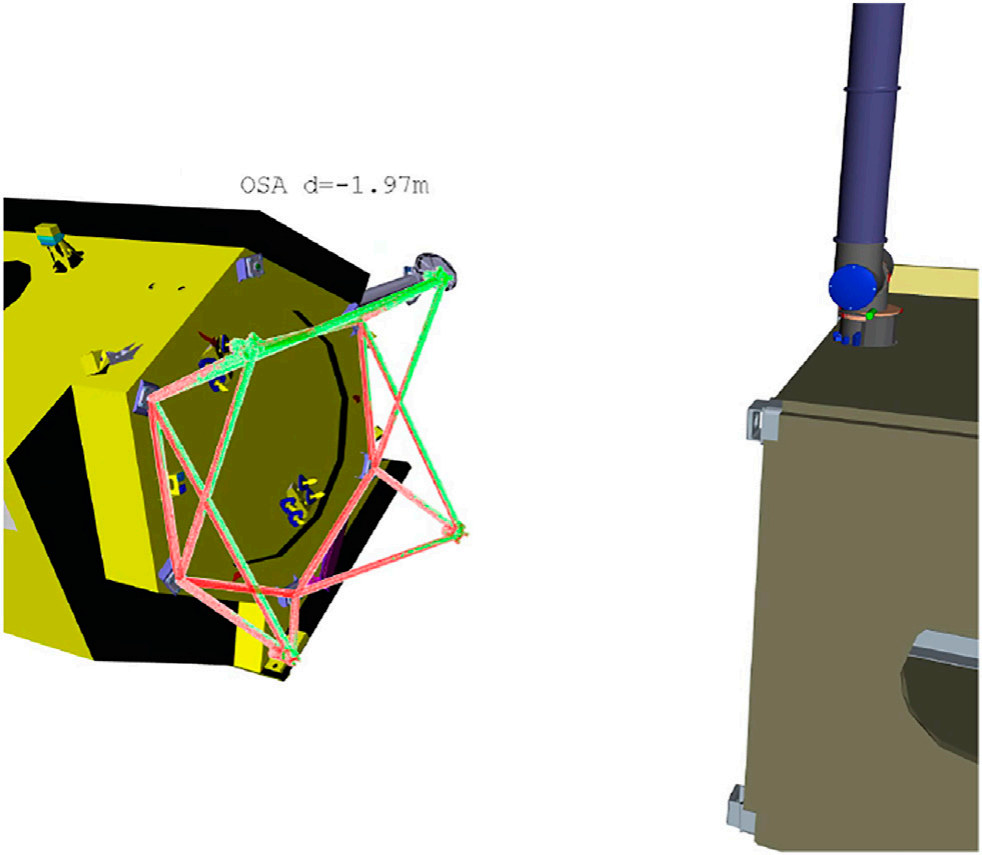
Online grasp selection for spacecraft docking using the plain reachability map for the manipulator. On the client satellite structure **(left)**, green points show the kinematically feasible grasp locations, while red shows unfeasible locations for this particular relative pose between servicer and client.

Reachability maps were previously used for selecting a grasp on the target satellite structure such that the docking maneuver is feasible, as presented in Porges et al. (2015). The capability map is used for obtaining a set of feasible grasps on the target structure. Figure 13 shows for instance the bar structure mounted on the TerraSAR-X satellite that should be grasped to pull the satellite for docking with the servicer.

Now, the method can be enhanced using the tools presented here such that the chosen grasp pose is robust to joint failures. The process described in (6) is followed, i.e., the failure diagram *D*(*ξ*) of the desired end-effector pose for the docking maneuver is computed. The *pfrs* workspace W(D(ξ)) using only ranges of failure diagram identified by *θ* is generated. Now, with W(D(ξ)) we can choose the best grasp location on the structure. Furthermore, the grasp selection can include the robustness-to-failure as a quality measure, utilizing the bin values *v*_*i*,*p*_ from $W_{f}$ . The grasp selected by maximizing the *v*_*i*,*p*_ value is the one that maximizes the *pfrs*. After a successful grasp, path planning procedures robust to failures can be applied, as described later in Section 5.2, to ensure that the docking maneuver is performed safely. Task planning modules are forced to only assume grasps within the safe workspace, thus guaranteeing post-failure docking feasibility.

**FIGURE 14:**
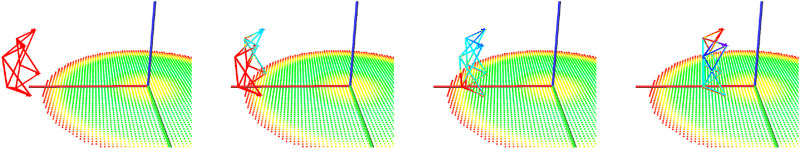
Grasp selection for fail-safe spacecraft docking. The figure depicts only the bar structure used for grasping (the structure mounted on the client satellite is shown in Figure 13), and shows how the grasp possibilities change depending on the target location with respect to the servicer. The color of the structure corresponds to the *v*_*i*,*p*_ value; blue areas are preferred for grasping. The grasp pose selected must lie in a region with high *v*_*i*,*p*_ across all the maneuver sequence.

The use of the combined failure map is depicted in Figure 14. The query poses are defined by pre-computed grasps on the target surface and passed through the *pfrs* workspace to obtain kinematically feasible and fail-safe grasp locations.

### 5.2 Path Planning

The necessary conditions for the existence of unidirectional and bi-directional fault-tolerant paths between two particular configurations were introduced in Section 4. Assuming that these conditions are met, a failure diagram can be used to generate a fault-tolerant motion and post-failure recovery path to reach the desired goal.

Given an initial and a desired goal, the first step is then verifying that there is a fail-safe tolerant path between them, as explained in Section 4. If this is the case, then a path can be obtained using any traditional path planning method, for instance, Rapidly-exploring Random Trees (RRT), which relies on random sampling of the C-space (LaValle et al., 2001). The only condition required to integrate the failure diagrams into the RRT planning approach is to use the bounds provided by the failure diagram (e.g. the bounds in Figure 10 bottom) for retrieving samples for the RRT process. In other words, each random joint position can only be retrieved from the joint ranges provided by the failure diagram for the desired end effector pose. This integration leads to a failure-tolerant RRT variant, as samples entering the RRT building process are inherently fail-safe.

On the onset of failure, the instantaneous joint configuration lies within the failure diagram, and the existence of a path to the goal pose is guaranteed. To obtain such path, the RRT planning process can proceed further by randomly sampling the remaining N_dof_ − 1 joints using still the joint ranges provided by the failure diagram; the locked joint does not change its value in the successive robot motions.

**FIGURE 15:**
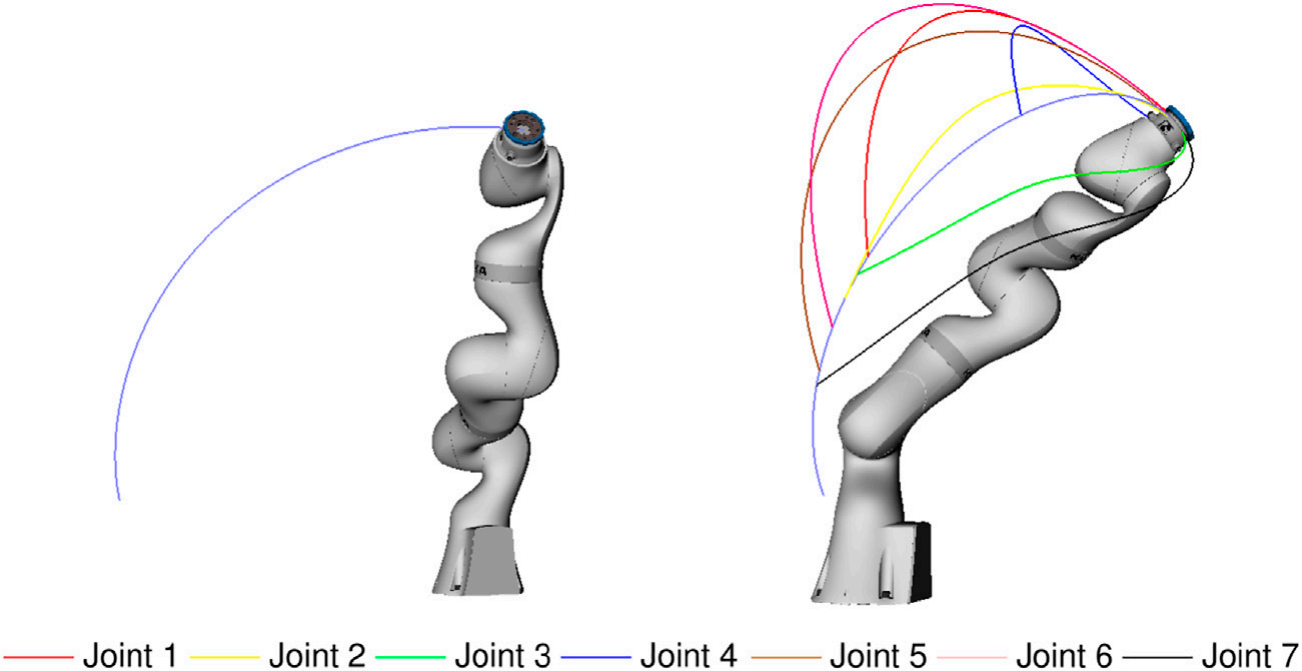
Failure-tolerant path. **Left:** nominal, failure-free path. **Right:** collection of paths to recover from random failures in each joint (as indicated by the corresponding color code).

An example of this approach is shown in Figure 15. A goal pose is defined, and a random feasible configuration is chosen as starting configuration. We could have used an RRT to plan the path between both poses; for simplicity, we used a simple joint interpolator to execute the path between the start and the goal configurations, respecting the joint bounds given by the failure diagram. This nominal path is visualized in Figure 15. Several random failures are simulated, one per each joint, during the path execution. For each one of them, we use an RRT planner for the remaining active joints to find an after-failure path to achieve the goal pose *ξ*, as shown in Figure 15 and in the attached video. As a result, the end-effector travels within the associated *pfrs* workspace depicted in Figure 12, and the end effector pose is achieved, within the tolerances given by the discretization used to build the required reachability maps.

### 5.3 Task Design and Base Positioning

A robotic manipulator mounted on a planetary rover allows tasks such as collection of soil samples, self-maintenance, or image-based diagnostics with an in-hand camera. Taking samples usually requires scratching and drilling on a rock or into the ground. Once samples have been collected, they need to be transported for devolatilization to a number of scientific instruments, and optionally to the storage area for later retrieval. Additional constrains on required end-effector poses can be given by actions such as visual self-inspection, where a predefined camera pose is desired.

The application of reachability maps for defining the mounting point of a manipulator on the rover structure was presented in Porges et al. (2015). Deciding the mounting point for the arm is a one-time task. However, if we use the failure maps considering the effect of collisions with the body on the resulting manipulator workspace, we can obtain a configuration that guarantees the execution of the tasks even in the case of a joint failure. The poses for the on-board instruments and for taking diagnostic pictures can be chosen based on bins with high values *v*_*i*,*p*_. Maximizing *v*_*i*,*p*_ also maximizes the *pfrs* workspace that spans from the associated poses. Bi-directional fail-safety for post-failure reachability can be guaranteed by using fused failure diagrams. The *pfrs* workspace can also be used to position the rover with respect to the task by following the inversion of reachability for base positioning introduced in Vahrenkamp et al. (2013). This workspace inversion is done only once, hence, it is not computationally demanding at the time of application. Thus, the method would provide a full 6-DoF base placement solution with respect to the task.

## 6 Discussion

This paper demonstrated how the concept of reachability maps is applied for analysis and design of fail-safe operations of robotic manipulators. Using failure maps, general dangerous zones can be identified both in task space (with *pfrs* workspaces) and C-space (with failure diagrams), and zones with high pre-failure redundancy can be selected for critical operations.

The reachability maps are based on a discretization of the workspace; its prediction accuracy was previously studied in Porges et al. (2014). In the case of failure diagrams, which is the crucial component for the computation of a failure-tolerant path, the chosen discretization must be considered carefully, as it also defines the tolerance allowed for reaching the desired end effector pose after a failure occurs. For the computation of failure diagrams, it is suggested to generate the reachability maps using forward kinematics, since it leads mostly to false positives on the boundary of the real workspace, as explained in detail in Porges et al. (2014). After the generation, the allowed ranges in the failure diagram (green cells in Figure 10 top) can be reduced by one bin on each side of the range to obtain conservative bounds for the fail-safe paths.

The computational requirements for the initial generation phase depend on the workspace volume (to the third power), as explained in Porges et al. (2014). In the case of KUKA iiwa, which was the robot used for the examples presented in this paper, approximately 4.5 CPU h (single core) were required for generating each one of the 2097 reachability maps with a locked joint, accounting for a total of approximately 9500 CPU h of a standard desktop computer. Generation of failure diagrams *D*(*ξ*) typically takes under 1 s, and spanning a fail-safe workspace W(D(ξ)) takes in the order of 5–10 min (volume-dependent). Although this computational cost is very large, this process is entirely performed offline, and the results are directly used for online queries or fail-safe path computations, as presented in Section 5.

The introduction of failure diagrams allows the search of a fault-tolerant path to reach one or multiple target poses. It can also be used to add constraints for established planning and execution methods, to find paths in case that a joint failure occurs. The planning time of fail-safe paths using RRTs extended with the failure diagrams is not strongly affected by using the failure diagrams, as they only modify the valid ranges for each joint used for the RRT sampling procedure. Therefore, the expected planning times are in the order of seconds, and depend on the particular situation. In general, a fail-safe path is found faster than an unconstrained path, since the search space is reduced by the boundaries imposed to get a fail-safe path.

The presented methods scale to kinematic chains with any number of DoF and joint types without further adaptations. Obstacles known at generation time can be incorporated into the computation of the initial reachability maps, while runtime obstacles can be incorporated according to the selected planning method.

While the presented methods and tools work for single joint failures, they are not easily extensible to two or more simultaneous failures due to the combinatorial increment of possible failure modes, which entails a much higher computational complexity. However, chances of two or more joint failures are slim, due to the redundancy used in the design of the robotic manipulators for space.

## Data Availability

The raw data supporting the conclusions of this article will be made available by the authors upon request.

## Footnotes

1
https://www.kuka.com/en-de/products/robot-systems/industrial-robots/lbr-iiwa
